# Se-Jin Lee, myostatin discoverer, elected to the National Academy of Science

**DOI:** 10.1186/2044-5040-2-11

**Published:** 2012-06-07

**Authors:** David J Glass, Bruce M Spiegelman

**Affiliations:** 1 Novartis Institutes for Biomedical Research, 100 Technology Square, RM4210, Cambridge, MA 02139, USA; 2 Dana-Farber Cancer Institute and Department of Cell Biology, Harvard Medical School, Boston, MA 02115, USA

**Keywords:** National Academy of Sciences, Skeletal muscle development, Myostatin

## Abstract

Se-Jin Lee was elected member to the National Academy of Sciences on 28 April 2012. Dr Lee is responsible for the discovery of myostatin, a critical regulator of skeletal muscle mass and function. He also determined the primary binding receptor for myostatin, and has characterized additional transforming growth factor–β family members acting in this pathway.

## Commentary

Professor Se-Jin Lee was elected on 28 April to the United States National Academy of Sciences for his research achievements, most notably the discovery of myostatin and its role in skeletal muscle maintenance.

Dr Lee is a Professor of Molecular Biology and Genetics at Johns Hopkins University. He holds an MD and PhD, and he joined Hopkins after a period as an associate at the Carnegie Institution for Science.

His first major paper in the area that led him on his most fruitful research path was published in *Nature* in 1994 [1]. In that study, Professor Lee reported on the discovery of several transforming growth facto (TGF)-β family members: growth differentiation factor (GDF)5, 6 and 7. Next, in 1997, Professor Lee and his co-authors Alexandra McPherron and Ann Lawler described the identification of myostatin, also known as GDF8, via RT-PCR, using degenerate primers from TGF-β family member homology domains [2]. In that same manuscript, they described the myostatin mouse knockout, and the dramatic skeletal muscle hypertrophy caused by deletion of myostatin, a phenotype that still manages to startle. A two- to three-fold increase in muscle mass was observed in the GDF8 null muscle [2]. Later that year, McPherron and Lee also reported that Piedmontese and Belgian Blue cattle, notable for their hypermuscularity, have naturally occurring disruptions of the myostatin locus [3].

Dr Lee went on to extensively study myostatin, and in subsequent papers demonstrated the loss of white fat that occurs upon induction of hypermuscularity by myostatin inhibition [4], and that administration of myostatin was sufficient to cause a phenotype reminiscent of cachexia [5]. He next showed that myostatin was regulated by a metalloprotease that activates it from a latent state [6], and identified the activin receptor type IIb receptor as one of the key receptors for myostatin [7]. This last paper highlighted an ongoing trend of first-author manuscripts for Dr Lee, a testament to his continued commitment to working at the bench.

Finally, Dr Lee has shown that other molecules in the TGF-β pathway, notably the activins and follistatin, also regulate muscle mass [8,9].

Dr Lee’s contributions include the demonstration of potential therapeutic benefits for myostatin inhibition. The precise clinical setting in which myostatin blockade might be beneficial has not yet been finally determined in humans, but it is at least clear that human null mutations for myostatin have a similar phenotype of hypermuscularity [10]. One potential area for blockade of this family of factors is cancer cachexia, as was demonstrated with an activin receptor IIb trap [11].

Dr Lee’s election to the Academy represents great recognition both for him and for the skeletal muscle field as a whole. His career is a testament to continued close study of a mechanism, leading to the initiation of an entirely new field of basic and biomedical research.

## References

[B1] Storm EE Huynh TV Copeland NG Jenkins NA Kingsley DM Lee S-J Limb alterations in brachypodism mice due to mutations in a new member of the TGF[beta]-superfamily Nature 1994 368 6472 639 643 10.1038/368639a0 8145850

[B2] McPherron AC Lawler AM Lee SJ Regulation of skeletal muscle mass in mice by a new TGF-beta superfamily member Nature 1997 387 6628 83 90 10.1038/387083a0 9139826

[B3] McPherron AC Lee SJ Double muscling in cattle due to mutations in the myostatin gene Proc Natl Acad Sci U S A 1997 94 23 12457 12461 10.1073/pnas.94.23.12457 9356471 PMC24998

[B4] McPherron AC Lee SJ Suppression of body fat accumulation in myostatin-deficient mice J Clin Invest 2002 109 5 595 601 11877467 10.1172/JCI13562 PMC150888

[B5] Zimmers TA Davies MV Koniaris LG Haynes P Esquela AF Tomkinson KN McPherron AC Wolfman NM Lee SJ Induction of cachexia in mice by systemically administered myostatin Science 2002 296 5572 1486 1488 10.1126/science.1069525 12029139

[B6] Wolfman NM McPherrin A Pappano W Davies M Song K Tonkinson K Wright J Zhao L Sebald S Grenspan D Activation of latent myostatin by the BMP-1/tolloid family of metalloproteinases Proc Natl Acad Sci U S A 2003 100 26 15842 15846 10.1073/pnas.2534946100 14671324 PMC307655

[B7] Lee S-J Reed LA Davies MV Girgenrath S Goad MEP Tomkinson KN Wright JF Barker C Ehrmantraut G Holmstrom J Regulation of muscle growth by multiple ligands signaling through activin type II receptors Proc Natl Acad Sci USA 2005 102 50 18117 18122 10.1073/pnas.0505996102 16330774 PMC1306793

[B8] Lee SJ McPherron AC Regulation of myostatin activity and muscle growth Proc Natl Acad Sci USA 2001 98 16 9306 9311 10.1073/pnas.151270098 11459935 PMC55416

[B9] Lee S-J Quadrupling muscle mass in mice by targeting TGFbeta signaling pathways PLoS One 2007 2 8 e789 10.1371/journal.pone.0000789 17726519 PMC1949143

[B10] Schuelke M Wagner KR Stolz LE Hubner C Riebel T Komen W Braun T Lee S-J Myostatin mutation associated with gross muscle hypertrophy in a child N Engl J Med 2004 350 26 2682 2688 10.1056/NEJMoa040933 15215484

[B11] Zhou X Wang LJ Lu J Song Y Kwak KS Jiao Q Rosenfeld R Chen Q Boone T Simonett WS Reversal of cancer cachexia and muscle wasting by ActRIIB antagonism leads to prolonged survival Cell 2010 142 531 543 10.1016/j.cell.2010.07.011 20723755

